# Assessing informal healthcare providers' knowledge of diagnosis and treatment of malaria and diarrhea: evidence from urban informal settlements in Southeast Nigeria

**DOI:** 10.3389/fpubh.2025.1556996

**Published:** 2025-03-12

**Authors:** Ifeyinwa Arize, Joy Ozughalu, Bernard Okechi, Chinyere Mbachu, Obinna Onwujekwe, Bassey Ebenso

**Affiliations:** ^1^Department of Health Administration and Management, Faculty of Health Sciences, College of Medicine, University of Nigeria, Enugu, Nigeria; ^2^Health Policy Research Group, College of Medicine, University of Nigeria, Enugu, Nigeria; ^3^Department of Psychology, Faculty of Social Sciences, University of Nigeria, Nsukka, Nigeria; ^4^Department of Community Medicine, Institute of Public Health, College of Medicine, University of Nigeria, Enugu, Nigeria; ^5^Leeds Institute of Health Sciences, University of Leeds, Leeds, United Kingdom

**Keywords:** malaria, diarrhea, informal health providers, urban slums, health services

## Abstract

**Background:**

Despite the availability of effective interventions, malaria and diarrhea continue to be leading causes of disease burden in Nigeria. Informal healthcare providers (IHPs) account for a significant proportion of health service providers in urban slums and may pose a challenge to service quality if they are untrained and unregulated. This study assessed IHPs' knowledge of the diagnosis and treatment of malaria and diarrhea.

**Methodology:**

A cross-sectional quantitative study was conducted in eight urban informal settlements (slums) in southeast Nigeria. Data were collected from 235 informal health providers using an interviewer-administered questionnaire.

**Results:**

The mean overall knowledge scores for malaria and diarrhea were 5.2 (95% CI: 4.3–6.1) and 5.4 (95% CI: 4.1–6.7), respectively, among the different IHPs. However, private medicine vendors (PMVs) and traditional birth attendants (TBAs) showed higher knowledge of treating malaria and diarrhea. Having more than 8 years of formal education and receiving on-the-job training had a statistically significant effect on adequate knowledge of malaria and diarrhea treatment.

**Conclusion:**

Institutionalizing and strengthening service delivery through appropriate training and support for IHPs can improve the quality of health service delivery in urban slums.

## Introduction

Communicable diseases, including malaria and diarrheal diseases, pose a significant challenge in many low- and middle-income countries (LMICs), such as Nigeria, as they disproportionately affect the poorest populations. Therefore, eliminating these diseases as public health concerns through appropriate prevention and control measures would greatly improve the health of the population.

Malaria remains a public health concern in Nigeria, with an estimated 68 million cases of malaria and ~194,000 malaria-related deaths occurring in 2021 (1). Furthermore, Nigeria is among the countries with the highest rates of malaria, with an estimated 25% of the global burden, leading households to incur catastrophic health expenditures due to the cost of treatment (2).

Similarly, diarrhea is a leading cause of childhood mortality and morbidity in developing countries such as Nigeria. The National Demographic Health Survey (30) reported that the prevalence of diarrhea in households with poor drinking water sources was slightly higher (16%) than in households with improved drinking water sources (12%) (2).

There has been rapid urbanization in Nigeria, which has led to increases in risk factors for many communicable diseases. In addition, rapid urbanization increases competition for scarce urban resources and underlines the need for policies that promote equitable access to resources (3). Poor sanitation and inadequate water supply predispose urban slum residents to diarrhea and create an environment conducive to breeding vectors for infectious diseases such as malaria. This problem is compounded by weak healthcare systems that lack sufficient infrastructure to cope with these burdens (4).

The growth rate of the urban population in Nigeria outpacing its economic growth, surpassing the capacity of the country's formal health system to adequately provide care, especially in urban slums (5). Therefore, it has become evident that the public sector alone cannot achieve the United Nations' Sustainable Development Goal 3 (SDG3) and universal health coverage. Collaboration and partnerships with private sector actors, including both formal and informal healthcare providers, are essential (6).

Informal healthcare providers (patent medicine vendors—PMVs and traditional birth attendants—TBAs, traditional bone setters, and herbal medicine practitioners) account for a significant proportion of healthcare providers in Nigeria, delivering care, especially in under-resourced communities (7). They are often the first point of care for common childhood and adult illnesses for those living in urban informal settlements in Nigeria (8). In communities with non-functional or inaccessible formal health systems, informal healthcare providers (IHPs) bridge the gap in access to healthcare services, even for households that would typically prefer to use formal (public) healthcare facilities (9, 10).

Widely available IHPs present opportunities to improve access to appropriate essential health services in underserved urban areas in many LMICs (7). However, IHPs lack the necessary formal training, proper regulation, and oversight (11, 12), which can compromise the quality and safety of the healthcare services they offer. While the contributions to healthcare delivery by IHPs in informal settlements are indisputable, the overarching concern of public health experts hinges on issues surrounding their knowledge and the quality of healthcare services they render.

Training IHPs to correctly recognize and manage common communicable diseases in urban slums (such as malaria and diarrheal diseases) will contribute to better healthcare for their clients. Several studies have highlighted the lack of understanding of malaria and diarrheal case management among informal providers (13–15). However, there is a paucity of evidence regarding the level of knowledge among informal health providers on the diagnosis and treatment of both malaria and diarrhea in urban informal settlements.

This is part of a larger study that involved informal providers in selected urban slums and formal providers in primary health centers serving these slums. This study provides new knowledge into the level of knowledge among informal health providers in slums regarding the diagnosis and treatment of communicable diseases, with a particular focus on malaria and diarrheal diseases, as well as the factors influencing such knowledge.

## Methods

### Study area

The study was conducted in eight urban slums informal settlements, each in Enugu and Anambra states in southeastern Nigeria. Enugu state had a population of ~4.4 million in 2019, while Anambra state had a population of 5.6 million in 2019 with an estimated 2.83% annual growth rate (1). Administratively, Enugu and Anambra states have 17 and 21 local government areas, respectively. Urban dwellers in both states are mostly civil servants, traders, transporters, or artisans.

It was a quantitative cross-sectional study. The study population was informal healthcare providers working in the purposively selected informal settlements. The eight informal settlements were purposively selected based on the size of the settlement, availability of different IHPs, and a functional primary health center close to the informal settlement. Areas with security challenges were excluded from the study. Enugu urban informal settlements included in the study were Afia Nine, Ugbo Oghe, Ngenevu, and Ikilike, while in Anambra state, the informal settlements sampled were Okpoko 4, Okpoko 5, Ibollo, and Prison Marine.

Data were collected from the heads of informal health facilities operating within the informal settlements using a pre-tested structured questionnaire. However, in their absence, any senior health provider in the facility was surveyed. Pre-testing of the instrument was performed in two slums not included in the study to assess the clarity and simplicity of the questionnaire items. The questionnaire was also reviewed by content and program experts to ensure the appropriateness of wordings and correct placement of items in the questionnaire. The survey was carried out between October and December 2022, with a computed minimum sample size of 256 providers including formal healthcare providers. The sample size was computed using the guidelines outlined in the Demographic and Health Survey (DHS) listing manual (32). A total of 235 informal health providers, including patent medicine vendors (PMVs), traditional birth attendants (TBAs), bonesetters, traditional healers, and herbal medicine dealers, participated in the survey, and their data were included in this study. Data from formal health providers, however, were excluded from this study.

The questionnaire was structured into the following seven sections: (1) background of the respondent (facility type, status in the facility, and training status); (2) knowledge, attitude, and practice (malaria and diarrhea); (3) treatment provision history; (4) motives and incentives; (5) challenges; (6) solutions for improving health provisions; and (7) determining the quality of malaria treatment as a case study, among others.

### Data analysis

Using SPSS (version 25), we calculated the frequency distributions of the key variables, including background characteristics of respondents and knowledge, diagnosis, and treatment of malaria and diarrhea. Using R statistical software (version 3.5.2), we calculated inferential statistics in the form of point estimates and 95% confidence intervals to draw conclusions from responses to the relevant questions. These statistics were presented as means and their associated 95% confidence intervals, as the questions had numerical responses. The relationship between the knowledge score and respondents' characteristics was analyzed.

Knowledge was summarized as composite scores, and two categories (adequate and inadequate knowledge) were generated. These two categories of knowledge are supported by the literature (16). The scores were added up to create a knowledge ranking for the aforementioned categories. The pooled scores of questions were classified into inadequate and adequate knowledge using median (50%) score values. Inadequate knowledge was labeled as ‘1', and adequate knowledge was labeled as ‘0'.

### Ethical consideration

Ethical approval for the study was obtained from the research ethics committees of the University of Nigeria Teaching Hospital, Enugu, and the University of Leeds. All the respondents gave informed verbal consent and were assured of total anonymity.

## Results

### Demographic characteristics of respondents

As shown in Table 1, patent medicine vendors were the most common informal health providers in the informal settlements, making up 73% and 72% of those surveyed in Anambra and Enugu states, respectively. It was found that 84.7% of the respondents were heads of the unit. On average, the respondents had up to 13.4 years of formal education and 76.2% received training for the job. More than 90% of the respondents provide services for both adults and children and have been providing healthcare services for more than 19 years. However, only 38% of the facilities are registered with the government agency/body (Table 1).

**Table 1:** Demographic characteristics of facilities.

***N* = 238**	**Combined**	**Anambra**	**Enugu**
	**235 (%)**	**115 (%)**	**120 (%)**
**Respondent's facility type**			
Patent medicine shop	152 (64.7)	73 (63.5)	79 (65.8)
Herbal home	41 (17.5)	21 (18.2)	20 (16.7)
TBA/nursing/maternity home	20 (8.5)	10 (8.7)	10 (8.4)
Bonesetters	18 (7.6)	9 (7.8)	9 (7.5)
Others (spiritual homes, etc.)	4 (1.7)	2 (1.7)	2 (1.7)
**Status of the respondent in the health facility**			
Head	198 (84.3)	107 (93.0)	91 (75.8)
Representative of head	37 (15.7)	8 (7.0)	29 (24.28)
Trained for the type of work	179 (76.2)	83 (72.2)	96 (80.0)
Years of formal education received (Mean, SD)	12.7 (4.09)	13.2 (3.01)	12.2 (4.8)
**Type of people that services are normally provided to**			
Children	12 (5.0)	4 (3.5)	8 (6.7)
Adults	9 (3.8)	89.6	1 (0.8)
Everybody	219 (91.3)	103 (89.6)	111 (92.5)
Length of time of service provision (in years; Mean, SD)	10.8 (11.2)	8.4 (9.3)	13.0 (12.33)
Is registered with Government Agency/body	92 (38.3)	26 (23.7)	63 (52.5)

**Figure 1:**
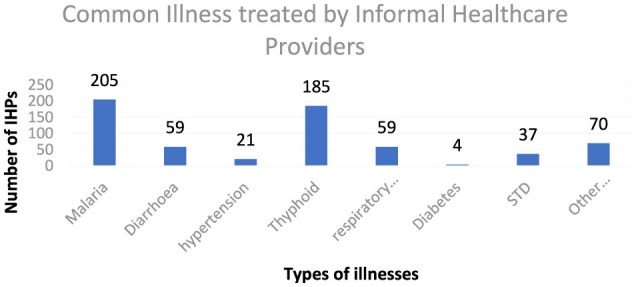
Common Illnesses treated by IHPs.

According to the findings in Figure 1, malaria accounted for the most common illness treated by informal providers, with over 90% of the IHPs identifying it as the most common illness they treated in their facilities. This is followed by typhoid, bone setting, respiratory tract infection, diarrhea, hypertension, and others. Across the two states, these common illnesses were also identified by the respondents as the same ones they treat in their facilities.

### Knowledge, diagnosis, and treatment of malaria

**Figure 2:**
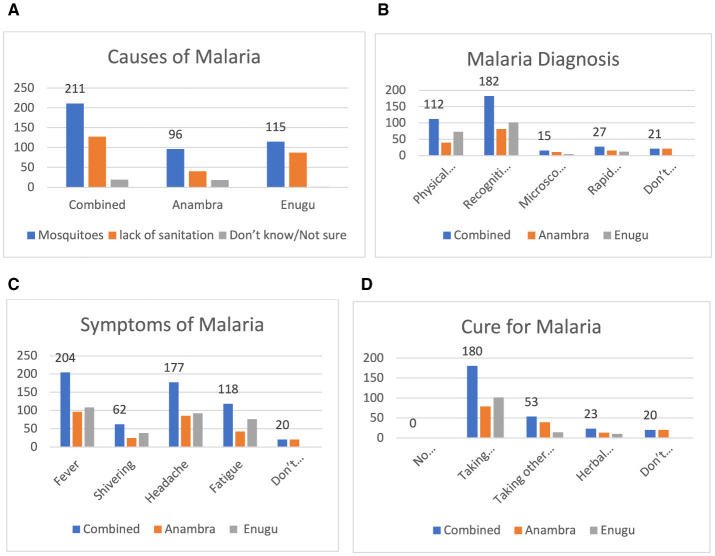
**(A)** Causes of malaria. **(B)** How malaria is diagnosed. **(C)** Responses for symptoms of malaria. **(D)** Responses on cure for malaria.

Figure 2A shows that 211 individuals (87.6% of the respondents) had the knowledge that malaria was caused by mosquitoes, and 131 individuals (52.4% of the respondents) attributed the increase in the number of malaria cases to poor sanitation. However, 20 persons (8.0% of the respondents) were not sure of what caused malaria. Figure 2B shows that for the diagnosis of malaria, 182 respondents (75.7%) were diagnosed by recognizing the symptoms and 112 respondents (47.7%) were diagnosed using physical examination. Only 27 (10.5%) and 15 (6%) of the respondents were diagnosed with malaria using a rapid diagnostic test or microscopic examination of blood slides, respectively. Figure 2C shows that ~204 (85%) and 177 (73.5%) respondents recognized fever and headache as key symptoms of malaria. As shown in Figure 2D, the majority of the respondents, 180 (74.8%), identified Artemisinin Combination Therapy as a treatment for malaria; however, 8.5% of the respondents either did not know or were unsure about the appropriate treatment.

### Knowledge, diagnosis, and treatment of diarrhea

**Figure 3:**
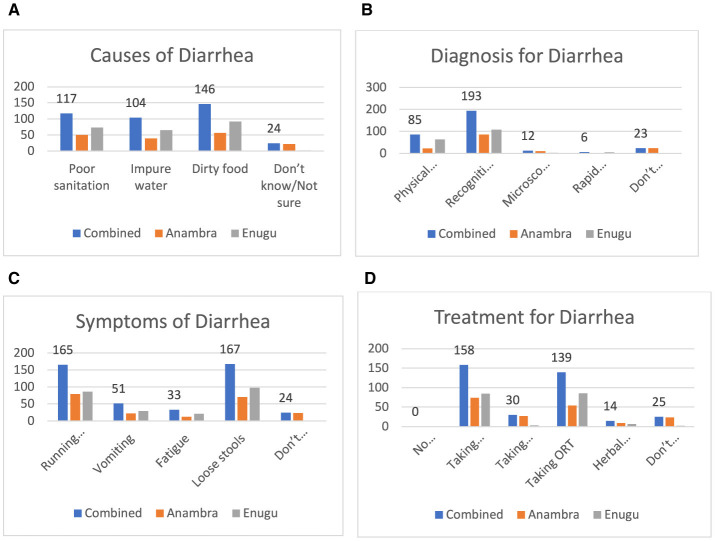
**(A)** Causes of diarrhea. **(B)** How diarrhea is diagnosed. **(C)** Symptoms of diarrhea. **(D)** Responses on cure for diarrhea.

Figure 3A shows that 146 (61.3%) and 117 respondents (50.8%) recognized that the causes of diarrhea were contaminated food and poor sanitation, respectively. However, 24 persons (10.0% of the respondents) were not sure of what caused diarrhea. Figure 3B shows that for the diagnosis of diarrhea, the majority of individuals, 193 (80.7%), were diagnosed by recognizing the symptoms, and 85 individuals (47.7%) were diagnosed using physical examination. Only 12 individuals (5% of the respondents) were diagnosed with diarrhea using microscopic examination of stools. Figure 3C shows that 167 (68.5%0 and 165 (68.3%) of the respondents identified the symptoms of diarrhea as loose stool and running stomach, respectively. Figure 3D shows that antibiotics and oral rehydration therapy (ORT) were identified as the treatment for diarrhea by 158 (65.5%) and 139 (57.6%) of the respondents, respectively.

**Figure 4:**
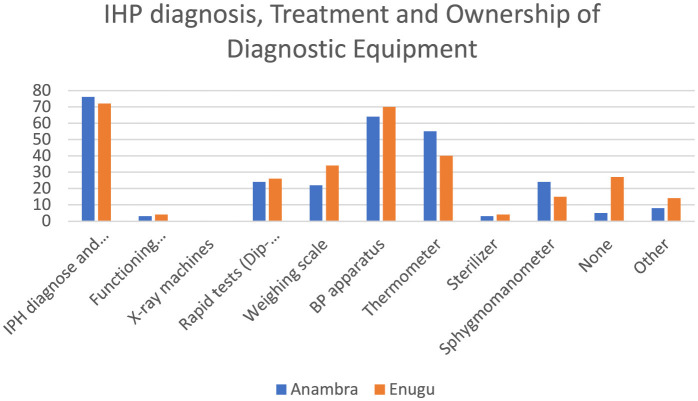
Available diagnostic equipment in the facility.

Figure 4 shows that more than half of the respondents were involved in both diagnosing illnesses and providing treatment. In terms of minimum standard equipment for health providers across both states, only ~50% had a blood pressure apparatus and 43% had a thermometer in their facilities. Less than 3% of the respondents had a functional microscope.

**Figure 5:**
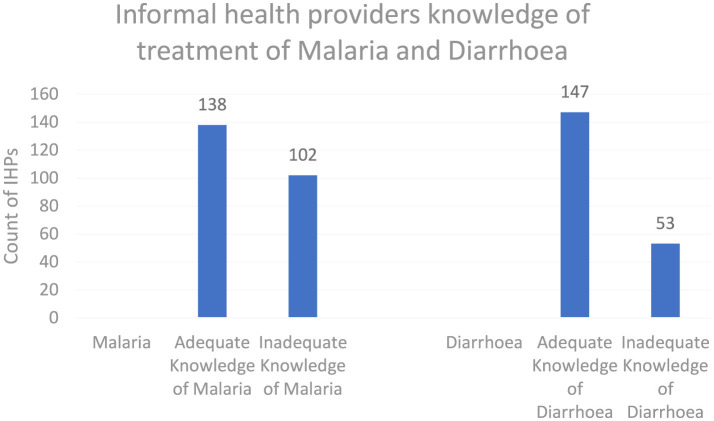
Knowledge frequency.

As shown in Figure 5, the knowledge score results, when categorized into adequate and inadequate knowledge levels for both malaria and diarrhea, showed that 57.5 and 61.2% of informal healthcare providers had adequate knowledge, respectively.

As shown in Table 2, a simple linear regression model estimated the mean knowledge scores for malaria and diarrhea at 95% CI to be 5.2 (4.3–6.1) and 5.4 (4.1–6.7), respectively. For these outcome variables, the model did not include any independent variable, as it only describes the central tendency of the knowledge scores and therefore does not provide any insights into the relationship between knowledge scores and other predictors.

**Table 2:** Association between knowledge score (for malaria and diarrhea) and demographic characteristics.

**Variables**	**Malaria**		***n*/total**	**Diarrhea**	
	**Mean 95% CI**	**Difference (95% CI)**		**Mean 95% CI**	**Difference (95% CI)**
Mean knowledge score	5.2 (4.3–6.1)			5.4 (4.1–6.7)	
**Years of education**					
(Ungrouped)	0.19 (0.01–0.21)			0.20 (0.12–0.29)	
**(Grouped)**					
< 8.67	3.63 (2.09, 5.16)	Reference		3.7 (2, 5.4)	Reference
8.67–12	5.80 (5.36, 6.25)	2.18 (1.04, 3.32)		6.2 (5.2–7.3)	2.51 (1.58–3.43)
Above 12	6.24 (5.79, 6.49)	2.52 (1.22, 3.81)		6.4 (5.4–7.3)	2.65 (1.46–3.83)
**Training status**					
No	2.70 (1.00–4.39)	Reference	184/240	2.55 (0.82, 4.29)	Reference
Yes	5.95 (5.55–6.35)	3.25 (1.85–4.65)	56/240	6.30 (5.42, 7.19)	3.75 (2.58–4.92)
**Status in the facility**					
Head	5.04 (4.07–6.02)	Reference	201/240	5.27 (3.96–6.58)	Reference
Representative	5.92 (5.39–6.46)	0.88 (0.10–1.66)	39/240	6.26 (5.18, 7.33)	0.99 (0.27–1.70)
**Facility type**					
Patent medicine vendors	5.98 (5.63, 6.33)	Reference	156/240	6.5 (4.21–7.31)	Reference
TBAs	6.00 (5.00, 7.00)	0.02 (−0.87, 0.90)	21/240	5.76 (5.80, 7.21)	−0.74 (−1.86, 0.39)
Herbal medicine	3.46 (2.10, 4.83)	−2.52 (−3.78, −1.24)	41/240	3.10 (1.12, 5.08)	−3.40 (−5.16, −1.65)
Bonesetter	1.83 (−1.26, 4.92)	−4.15 (−7.07, −1.23)	18/240	1.78 (−0.97, 4.52)	−4.72 (−6.98, −2.47)
Others	2.75 (−3.23, 8.73)	−3.23 (−8.95, 2.49)	4/240	4.0 (−3.30, 11.30)	−4.25 (−8.49, 0.05)
**Years of service provision**					
(Ungrouped)	0.00 (−0.00 to 0.01)			0.00 (−0.00, 0.01)	
**(Grouped)**					
< 4 years	5.35 (4.20, 6.47)	Reference		5.49 (3.67, 7.32)	Reference
4–10 years	4.87 (3.84, 5.90)	−0.46 (−0.97, 0.05)		5.09 (3.71, 6.46)	−0.41 (−1.34, 0.53)
Above 10 years	5.35 (4.78, 5.92)	0.02 (−0.77, 0.80)		5.72 (4.88, 6.55)	−0.22 (−1.10, 1.55)

Respondents with more than 8 years of education had higher knowledge scores on malaria than those with fewer years of education. Specifically, those with 8.67–12 years of education had a mean score of 5.80 (95% CI: 5.36, 6.25), which was 2.18 points higher (95% CI: 1.04, 3.32) than the reference group with < 8.67 years of education. Respondents with more than 12 years of education had an even higher mean score of 6.24 (95% CI: 5.79, 6.49), showing a difference of 2.52 points (95% CI: 1.22, 3.81) compared to the reference group.

Training status also significantly influenced malaria knowledge scores. Respondents without training had a mean score of 2.70 (95% CI: 1.00, 4.39), while those with training had a mean score of 5.95 (95% CI: 5.55, 6.35), indicating a substantial increase of 3.25 points (95% CI: 1.85, 4.65).

In terms of facility status, heads of facilities had a mean score of 5.04 (95% CI: 4.07, 6.02), whereas representatives had a mean score of 5.92 (95% CI: 5.39, 6.46), with a difference of 0.88 points (95% CI: 0.10, 1.66).

Facility type significantly impacted the knowledge scores. Patent medicine vendors had a mean score of 5.98 (95% CI: 5.63, 6.33), while those practicing herbal medicine and bone setting had lower mean scores of 3.46 (95% CI: 2.10, 4.83) and 1.83 (95% CI: −1.26, 4.92), respectively. These differences were −2.52 points (95% CI: −3.78, −1.24) and −4.15 points (95% CI: −7.07, −1.23) when compared to patent medicine vendors.

Years of service provision, whether grouped or ungrouped, showed minimal impact on knowledge scores. Respondents with < 4 years of service had a mean score of 5.35 (95% CI: 4.20, 6.47), while those with 4–10 years had a mean score of 4.87 (95% CI: 3.84, 5.90), with a difference of −0.46 points (95% CI: −0.97, 0.05). Respondents with more than 10 years of service had a mean score of 5.35 (95% CI: 4.78, 5.92), showing a negligible difference of 0.02 points (95% CI: −0.77, 0.80).

### Diarrhea

Respondents' knowledge scores on diarrhea were also influenced by their years of education. Those with 8.67–12 years of education had a mean score of 6.2 (95% CI: 5.2, 7.3), which was 2.51 points higher (95% CI: 1.58, 3.43) than the reference group with < 8.67 years of education. Similarly, respondents with more than 12 years of education had a mean score of 6.4 (95% CI: 5.4, 7.3), showing a difference of 2.65 points (95% CI: 1.46, 3.83) compared to the reference group.

Training status had a marked effect on knowledge scores for diarrhea. Respondents without training had a mean score of 2.55 (95% CI: 0.82, 4.29), while those with training had a mean score of 6.30 (95% CI: 5.42, 7.19), reflecting an increase of 3.75 points (95% CI: 2.58, 4.92).

Facility status showed that heads of facilities had a mean score of 5.27 (95% CI: 3.96, 6.58), while representatives had a mean score of 6.26 (95% CI: 5.18, 7.33), with a difference of 0.99 points (95% CI: 0.27, 1.70).

Knowledge scores varied significantly with the type of facility. Patent medicine vendors had a mean score of 6.5 (95% CI: 4.21, 7.31). In contrast, those practicing herbal medicine and bone setting had lower mean scores of 3.10 (95% CI: 1.12, 5.08) and 1.78 (95% CI: −0.97, 4.52), with differences of −3.40 points (95% CI: −5.16, −1.65) and −4.72 points (95% CI: −6.98, −2.47) compared to patent medicine vendors.

Years of service provision, whether grouped or ungrouped, had a minimal effect on diarrhea knowledge scores. Respondents with < 4 years of service had a mean score of 5.49 (95% CI: 3.67, 7.32), while those with 4–10 years had a mean score of 5.09 (95% CI: 3.71, 6.46), with a difference of −0.41 points (95% CI: −1.34, 0.53). Respondents with more than 10 years of service had a mean score of 5.72 (95% CI: 4.88, 6.55), with a difference of −0.22 points (95% CI: −1.10, 1.55).

## Discussion

The findings that informal health providers deliver healthcare for common illnesses for both adults and children were expected from the large numbers in the informal settlements. Our findings are consistent with the evidence from other studies that informal health providers are a crucial source of healthcare for urban poor populations and communicable diseases, which include malaria and diarrhea (17).

The findings on the availability of diagnostic equipment showed that the majority of IHPs lack basic equipment for providing care in their facilities. Therefore, they rely mostly on patient history and symptoms to provide care. The findings are similar to evidence from Bangladesh that showed that in addition to knowledge gaps, IHPs lacked diagnostic tools and medicines to deliver services effectively (18). The implications are that they may provide inaccurate diagnoses and delayed treatments, impacting the quality of care.

The findings on the method of diagnosis showed that the primary way informal providers diagnose malaria is by recognition of symptoms. This is similar to the findings by Ayandipo et al. (31) in Nigeria, where over 85% of health providers exhibited very poor malaria diagnostic practices. This differs from the WHO case management protocol for malaria, which recommends microscopy, RDTs, or both for the diagnosis of malaria. In this study conducted in Enugu and Anambra states in Nigeria, only a few IHPs diagnosed malaria using a rapid diagnostic test or microscopic examination of blood slides. It implies that they either do not recognize the need or that they lack the competencies and equipment for correct diagnosis (19). Whichever is the case, improper diagnosis of malaria can lead to antimalarial drug resistance arising from inappropriate treatment.

Furthermore, antimalarial drug resistance could be a result of inappropriate treatment and the use of substandard drugs. The use of inappropriate treatment and substandard drugs may be an important contributor to the emergence and spread of antimicrobial resistance (AMR) (20, 21). AMR is a global public health problem, with resistance to antibiotics causing an estimated 1.3 million deaths in 2019 (22).

Training on the job, years of education, and the type of facility had a significant impact on knowledge regarding the treatment of malaria and diarrhea. Furthermore, those who received on-the-job training, had more years of education, and worked as PMV or TBAs demonstrated better knowledge of treating malaria and diarrhea. This could imply that they may have an innate ability to understand and internalize the information received during training, which may translate into improved access to quality care and better patient outcomes. These findings are consistent with a previously published study that showed a positive association between training and knowledge of non-communicable diseases among IHPs (23).

However, evidence shows that deploying training alone may have only a modest impact on performance (24). Therefore, it is recommended that supportive supervision, job aides, and both financial and non-financial incentives be incorporated to encourage good practices. These combined strategies have been found to be effective in improving the performance of IHPs (25).

There may be an urgent need to provide support and appropriate training strategies for informal health providers to ensure quality healthcare, especially for communicable diseases. Training and providing support has been found to be one of the ways of improving knowledge of diagnosis and treatment of any disease (26). We therefore recommend the use of appropriate training, job aides, and support for informal health providers to improve their diagnosis and treatment of malaria and diarrhea.

Furthermore, although IHPs have different educational backgrounds and practices, the availability of mobile technologies could allow for context-specific information (in the local language) to be made accessible to them. This would provide up-to-date knowledge of malaria and other common ailments. Digital technologies have been found to promote health literacy and empowerment by providing clear, reliable, and accessible information (27, 28) in a fun and effective way (29). This could facilitate improvements in the provision of quality health services by IHPs.

The study used a small sample size and therefore may not be generalizable; however, the inclusion of different types of informal providers may have made the study more robust. The use of only quantitative methods is also a limitation as there could be bias in the respondent's answer, and it cannot be used to infer certain factors. However, the study has provided valuable information on informal health providers.

Informal health providers' knowledge of the diagnosis and treatment of malaria and diarrhea is inadequate. Strengthening service delivery through appropriate training and provision of health literacy tools for the different types of informal health providers has the potential to improve health service delivery in urban informal settlements. Ensuring quality service delivery through intervention is essential for the achievement of SDG3 (good health and wellbeing) and SDG4 (quality education). Providing quality education will, in turn, lead to improved quality of service delivery.

## Data Availability

The raw data supporting the conclusions of this article will be made available by the authors, without undue reservation.
